# Role of Workplace Spirituality, Empathic Concern and Organizational Politics in Employee Wellbeing: A Study on Police Personnel

**DOI:** 10.3389/fpsyg.2022.881675

**Published:** 2022-04-29

**Authors:** Shreshtha Yadav, Trayambak Tiwari, Anil Kumar Yadav, Neha Dubey, Lalit Kumar Mishra, Anju L. Singh, Payal Kapoor

**Affiliations:** ^1^Department of Psychology, Banaras Hindu University, Varanasi, India; ^2^Department of Psychology, Rajendra College, Jai Prakash University, Chapra, India; ^3^Department of Psychology, Indira Gandhi National Tribal University, Amarkantak, India; ^4^Department of Psychology, Vasanta Kanya Mahavidhyalaya, Banaras Hindu University, Varanasi, India; ^5^Independent Researcher, Prayagraj, India

**Keywords:** mindfulness, meaningfulness, employee wellbeing, empathetic concern, organizational politics, moderated mediation analysis

## Abstract

Employee wellbeing as a central aspect of organizational growth has been widely regarded and accepted. Therefore, a considerable growth in the number of researches focusing on employee wellbeing has been comprehended in recent years. Employee wellbeing characterizes the individual’s own cognitive interpretation of his/her life at work. The present study made an attempt to examine how workplace spirituality, empathic concern and organizational politics influences employee wellbeing. It was hypothesized that empathic concern mediates the relationship between workplace spirituality and employee wellbeing while organizational politics act as a moderator in this relationship. A survey was conducted on 253 employees working in Uttar Pradesh Police department (Uttar Pradesh, India). The results obtained revealed that workplace spirituality, empathic concern and employee wellbeing carries a positive association among them whereas these variables were found to be negatively correlated with organizational politics. Results also depicted that empathic concern significantly mediates between workplace spirituality and employee wellbeing. Further, moderated mediation analysis confirmed employee wellbeing as a function of workplace spirituality, empathic concern and organizational politics. The present study has put forward several practical implications for business practitioners and research directions for academicians, emphasizing upon the need to investigate the comprehensive impact of employee wellbeing in organization and the society as a whole.

## Introduction

Alike development (physical, psychological), identity, social relationship, work (job) is also a fundamental facet of human life. For an individual, having a job is essential to accomplish his economic freedom as well as to fulfill his physiological, social and psychological needs. Therefore, we all put a significant amount of our strength to find a suitable job and thereby gets involved to maintain its status in the society. Generally, a person spends almost one-third of a day at the workplace. Nevertheless, in the Indian context, the working hours or hours on work-related tasks are higher than one-third of a day (Periodic Labour Force Survey, 2021). Considering the working hours the Indian workforce spends at the workplace, it is apparent that their life at work significantly impacts their overall wellbeing. Therefore, it may be assumed that having a good working environment is a crucial determinant of an individual’s overall life satisfaction and wellbeing. This implies that employers should focus and make sincere efforts to promote employees’ wellbeing at the workplace to maintain a motivated and competent workforce. As this will consequently help in enhancing the overall wellbeing of the employees, along with their family members and the society at large.

### Employee Wellbeing

Work-related issues [e.g., work stress, organizational politics (OP), and interpersonal relationships] often trigger various other problems for the employees those consequently affect their family and the society. Employee wellbeing (EWB) is one of the topics of significance in the area of organizational behavior and management. Organizations develop and implement different training and other programs focused on enhancing employee wellbeing. The major reason for taking this initiative generally lies in the consequences associated with employee wellbeing, which include better work performance, low turnover intentions, less injuries, high work motivation, job engagement and low absenteeism (Keyes, 2005; Lyubomirsky et al., 2005; Boehm and Lyubomirsky, 2008; Bakker, 2015). Before conceptualizing the concept of employee wellbeing, it is important to understand the meaning of wellbeing. There are plentiful explanations of wellbeing in the literature. For instance, Diener (1984) defined wellbeing as an individual’s own overall evaluation of his/her work, life situations, surroundings and emotional experiences. Grounded on his views, employee wellbeing can be defined as employee’s comprehensive subjective discernment of satisfaction and positive attitude toward their work.

Extensive literature review revealed that the concept of wellbeing has been conceptualized in different ways. However, there are two major perspectives that include: (a) hedonic perspective (b) eudemonic perspective. These two perspectives are pointedly distinct in nature. Hedonic perspective consider happiness as an indicator of wellbeing; while the eudemonic perspective believes in character virtues like self-actualization (Ryan and Deci, 2001; Dewe and Cooper, 2012). Further, Diener et al. (1985) maintained that the hedonic perspective advocated the importance of three dimensions of wellbeing, which included having a positive mood, absence of negative mood and life satisfaction. Whereas the eudemonic perspective lays emphasis on an individual’s character, means to achieve happiness and growth as essential components of wellbeing. Ryff (1989, 1995) reported that an individual can attain wellbeing by engaging in six different activities, which included: life purpose, personal growth, self-acceptance, mastery, autonomy and positive relatedness.

Initially, it was believed that both hedonic and eudemonic perspectives were mutually exclusive, but lately scholars pointed out that for a better understanding of wellbeing we need to incorporate the characteristics of both. Completely relying on single (any one) of these perspectives seems insufficient to explain the meaning of wellbeing. As Fisher (2014) pointed out that wellbeing is a very complex concept to understand and explore; it can only be understood if it is taken as a multidimensional concept that includes the unique aspects of both perspectives.

The present study follows the argument that wellbeing is a multidimensional concept, and aspects of both perspectives (hedonic and eudemonic) should be considered while operationalizing employee wellbeing. Therefore, in this study employee wellbeing is operationalized as an amalgamation of hedonism and eudemonia perspectives which includes the dimensions of happiness and self-esteem.

### Spirituality at Workplace

The concept of spirituality at the workplace (SW) has recently gained popularity among business scholars and practitioners. Commonly it is believed that spirituality is another name for the concept of religiosity, but it is not a comprehensive reality. There may be certain similarities in some aspects of religiosity and spirituality, but spirituality as a concept is widely different from religiosity (Milliman et al., 2003). The concept of religiosity encompasses a relatively permanent system and pattern of faith or belief of a group of people (Newberg, 2014; Afsar and Rehman, 2015). On the other hand, the concept of spirituality discusses connectivity among individuals, meaning in life, purpose, integration, growth, truth and mindfulness (Myers, 1990; Harrington et al., 2004; Beheshtifar and Zare, 2013). Therefore, the concept of spirituality discusses beyond the terrestrial and institutional aspects of religiosity (Petchsawang, 2008).

Workplace spirituality has been tried to explain incorporating various aspects including the dimensions such as inner life connection, sense of community, connectedness, compassion, transcendence, mindfulness and meaningful work (Mitroff and Denton, 1999; Jurkiewicz and Giacalone, 2004; Kinjerski and Skrypnek, 2006). In the present study, workplace spirituality is defined as an inner sense of connectedness and completeness with their work. This inner sense of connectedness and completeness is a consequence of an interaction between two characteristics of the workplace named mindfulness and meaningful work.

The concept of mindfulness is used to express the state of inner awareness and consciousness (Phan et al., 2020). This implies that the individual is consciously aware of his/her present thoughts and actions. It considers active monitoring of actions only through the lenses of the present without any disruptions from the past experiences or expectations from the future (Heaton et al., 2004). A person adorned with the quality of mindfulness lives his/her life in the present; he/she does not behave like a slave of past experiences and does not ruin his/her state of wellbeing with uncertainties of the future (Walach et al., 2006; La Torre et al., 2020). Another important representative of mindfulness is owning freedom from external distractions. A mindful person can get rid of different worldly distractions that deviate the person from putting all his/her efforts into present activities (Brown and Ryan, 2003).

Meaningful work is another essential feature of workplace spirituality. It can be defined as an individual’s belief that his/her work has a significant impact on one’s own life as well as other members of society. Duchon and Plowman (2005) argued that meaningful work has a more positive impact on performance than external rewards (materialistic benefits). Furthermore, meaningful work motivates, energizes and gives a sense of meaning in the life of employees (Overell, 2008).

### Organizational Politics

As far as the availability of resources is concerned, a similarity can be observed between the functioning of organizations and the societies. Members of organizations often struggle to acquire maximum resources similar to the individuals of the certain societies. This struggle leads to interpersonal conflict and engagement into various activities aimed to secure maximum resources without evaluating the righteousness of these activities (Molm, 1997; Hochwarter, 2012). Fundamentally, using inappropriate strategies to maximize personal benefits is a core ingredient of organizational politics (Ferris et al., 1989; Kacmar and Baron, 1999). However, in extended form, organizational politics can be understood as employees’ egocentric and morally illegitimate behaviors (such as exploiting organizational resources and taking advantage of other employees) that are envisioned to accomplish personal interests (Mintzberg, 1985; Ferris and Kacmar, 1992). Therefore, organizational politics is often considered as a negative function and challenge to the collective objectives of the organization (Block, 1988).

Affective event theory (Weiss and Cropanzano, 1996) supports the idea that various events at the workplace cause emotional disturbance in the life of employees. Further, this emotional disturbance damages not only the physical health but psychological health and overall wellbeing of the employees. Based on effective event theory, Thiel et al. (2014) advocated that different incidents of organizational politics stimulate the affective state of employees and result in intense emotional responses. Though enough literature has provided evidence of the negative consequences of organizational politics but its impact on employee wellbeing is not explored in depth. Given the meaning and motive of organizational politics, it can be concluded that organizational politics has a negative influence on employee wellbeing. Therefore, the present study focused on how organizational politics influences the relationship between workplace spirituality and employee wellbeing.

### Empathic Concern

Empathic concern (EC) is one of the most discussed concepts in the area of social and organizational psychology. Initially, it was believed that empathic concern is a multidimensional concept (Smith, 1759; Watson and Tellegen, 1985). However, in later years, scholars argued that empathic concern is a unidimensional construct, as its dimensions share a significant commonality in operationalization and measurement (Hoffman, 1977, 2000). Considering this, empathy can be defined as an individual’s ability to experience the emotional strain of others and express concern for them (McNeely and Meglino, 1994). Lazarus (1991, 1999) extended the meaning of empathy and explained it as an individual’s capacity to place himself/herself in the situation of others and experience their sufferings. Further, Eisenberg (2000) and Cliffordson (2002) advocated that empathy encompasses cognitive as well as emotional processes. Cognitive processes include perceiving, examining, understanding and comprehending the situation of others, while emotional processes include sharing the feelings and emotions of others (Davis, 1983; Decety and Jackson, 2004).

Empathic concern is an important tool to make people abide with the social context (Muller et al., 2014). It also assist in facilitating social cognition, personal bonding, interpersonal engagement, healthy relationships and honest apprehension for other’s wellbeing (Davis, 1990; Reynolds and Scott, 2000; Scott et al., 2010). The impact of empathic concern on wellbeing in social context is well discussed (Diener et al., 1999; Haxby et al., 2000). However, in organizational context the role of empathic concern in relation to workplace spirituality and employee wellbeing remains somewhat unattended. This warrants a need to explore this aspect of empathic concern.

On the basis of review of literature a research gap was established and present study was aimed at exploring these issues. Following hypotheses were formulated for the study:

H_1_: There will be a positive association between workplace spirituality and employee wellbeing.

H_2_: Organizational politics will moderate the relationship between workplace spirituality and employee wellbeing; and would be negatively associated with workplace spirituality.

H_3_: Organizational politics will moderate the relationship between empathic concern and employee wellbeing.

H_4_: Empathic concern will mediate the relationship between workplace spirituality and employee wellbeing.

H_5_: Empathic concern would be positively associated with workplace spirituality and employee wellbeing and negatively associated with organizational politics.

## Methods

The present study was designed to explore whether empathic concern mediates the relationship between workplace spirituality and employee wellbeing. In addition, whether organizational politics moderate the impact of workplace spirituality and empathic concern on employee wellbeing was also explored (Supplementary Figure 1).

### Sampling and Participants

In this research, a survey was conducted on police personnel of Uttar Pradesh Police Department (UPPD, India). Convenient sampling (a method of Non-probability sampling) was used to select the participants for data collection. For the survey, 300 employees of UPPD, India were contacted. However, only 253 participants have responded and completed the survey. Thus, responses from 253 participants (male = 177, female = 76) were analyzed. The participants’ age ranged from 22 to 57 years (Mean = 37.40, S.D. = 9.29); and their work experience ranged from 0.2 to 26 years (Mean = 10.10, S.D. = 8.74).

### Procedure

Before initiating the data collection for the study, Uttar Pradesh Police Service departments, located in different districts of Uttar Pradesh, were approached. Prior appointments were fixed with the concerned authorities. They were informed about the study’s objectives and the procedure of data collection in detail. Questionnaires were administered among the participants after attaining their consent. Overall, 300 sets of questionnaires were distributed to the employees of the police department located in different districts of Uttar Pradesh. Out of 300 employees, 278 employees provided their consent for the data collection and 253 completed the questionnaires.

### Measures

#### Empathic Concern

An adapted version of a subscale from the interpersonal reactivity index (Davis, 1980) was used to measure empathic concern. The interpersonal reactivity index (IRI) has four subscales: perspective-taking, fantasy, empathic concern, and personal distress. The subscale of empathic concern is made of seven items, and the responses were noted on a seven-point Likert scale. The Cronbach’s alpha of this subscale was estimated as 0.94 in the present study.

#### Workplace Spirituality

A scale intended to measure workplace spirituality in an Asian context (Petchsawang and Duchon, 2009) was used to measure workplace spirituality. In accordance with the operationalization of workplace spirituality, two dimensions (mindfulness and meaningful work) were considered in the present study. Internal reliability of this scale was found as Cronbach’s alpha = 0.96.

#### Organizational Politics

A six-item scale developed by Yadav (2019) was employed to measure organizational politics. Responses were recorded using Likert type seven-point scale, and Cronbach’s alpha of this scale was found to be 0.95.

#### Employee Wellbeing

Employee wellbeing has been measured by Well-Being Manifestations Measure Scale (Massé et al., 1998). This measure includes two dimensions namely self-esteem and happiness. These two dimensions encompass both perspectives (hedonic and eudemonic) of employee wellbeing. A seven-point Likert type scale was used to record the participant’s responses. In the present study reliability of this measure was recorded as Cronbach’s alpha = 0.97.

## Results

### Descriptive Statistics, Reliability and Correlational Analysis

Table 1 illustrates the descriptives (Mean, S.D.), reliability and correlation coefficients among research variables. Aligned with the research hypotheses, workplace spirituality was found to be positively correlated with empathic concern (*r* = 0.55^**^) and employee wellbeing (*r* = 0.36^**^); similarly empathic concern was found to be positively associated with employee wellbeing (*r* = 0.37^**^). Table 1 shows that organizational politics owns a negative association with workplace spirituality (*r* = −0.23^**^), empathic concern (*r* = −0.17^**^) and employee wellbeing (*r* = −0.48^**^).

**TABLE 1:** Descriptive statistics, Cronbach’s alpha and correlation coefficients among variables.

	Mean	S.D.	WS	EC	OP	EWB
WS	57.05	18.26	*0*.*96*			
EC	31.84	10.48	0.55**	*0.94*		
OP	18.91	8.91	−0.23**	−0.17**	*0*.*95*	
EWB	38.88	14.84	0.36**	0.37**	−0.48**	*0.97*

N = 253; Cronbach’s alpha in italics; **p < 0.01.

### Moderated Mediation Analysis

**FIGURE 1:**
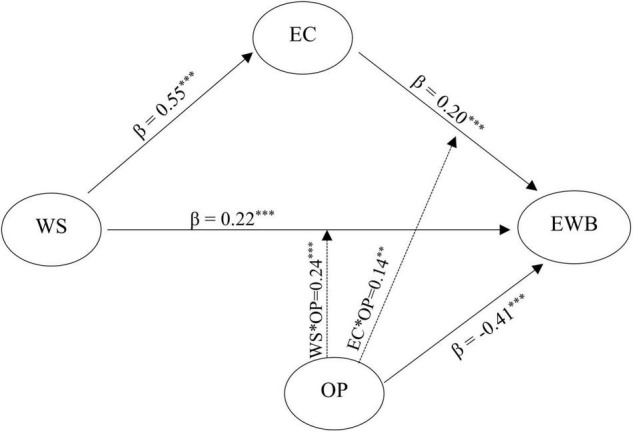
Mediating effect of empathic concern between workplace spirituality and employee wellbeing as a function of organizational politics. Moderated mediation model (all coefficients are standardized). Here ^**^ means *p* < 0.01 and ^***^ means *p* < 0.001.

Figure 1 demonstrates, the standardized regression coefficients between workplace spirituality and empathic concern (β = 0.55^***^); empathic concern and employee wellbeing (β = 0.20^***^); workplace spirituality and employee wellbeing (β = 0.22^***^); organizational politics and employee wellbeing (β = −0.41^***^) and all these coefficients were found to be statistically significant. Table 2 illustrates the importance of each predictor in forecasting employee wellbeing. Table 2 also shows that all predictor variables together could predict thirty percent variance (*R*^2^ = 0.30) in probability of employee wellbeing. It also depicts the conditional indirect effect of empathic concern on the relationship between workplace spirituality and employee wellbeing using organizational politics as moderator. Conditional indirect effect was found to be strongest when the perception of organizational politics was low (−1 S.D.; indirect effect = 0.189; LLCI = 0.081; ULCI = 0.302 at 95%). In case of average perception (Mean) of organizational politics, indirect effect was found lower than low perception of organizational politics but statistically significant (Mean; indirect effect = 0.109; LLCI = 0.038; ULCI = 0.187 at 95%). Whereas, indirect effect was found lowest and statistically not significant if the perception of organizational politics was noted high (+1 S.D.; indirect effect = 0.030; LLCI = −0.056; ULCI = 0.122 at 95%).

**TABLE 2:** Testing the mediating effect of EC and moderated-mediating effect of OP and EC.

Employee wellbeing as outcome					

Predictors	β	t	p	LLCI	ULCI
WS	0.22	3.55	0.000	0.095	0.334
EC	0.20	3.43	0.000	0.085	0.314
OP	–0.41	8.69	0.000	–0.512	–0.323
WS × OP	0.24	4.26	0.000	–0.353	–0.120
EC × OP	0.14	2.66	0.008	–0.251	–0.037
R^2^	0.30				
F	109.31***				

**Conditional direct effect WS on EWB**				

	**Effect**	**Boot SE**	**Boot LLCI**	**Boot ULCI**

−1 S.D	0.456	0.09	0.267	0.646
Mean	0.215	0.06	0.095	0.334
+ 1 S.D.	–0.026	0.06	–0.158	0.104
**Conditional indirect effect of WS on EWB through EC**				
−1 S.D	0.189	0.05	0.081	0.302
Mean	0.109	0.03	0.038	0.187
+ 1 S.D.	0.030	0.04	–0.056	0.122

**Index of Moderated Mediation**				

	**Index**	**Boot SE**	**Boot LLCI**	**Boot ULCI**

	–0.079	0.03	–0.145	−0.013

***p < 0.001.

**FIGURE 2:**
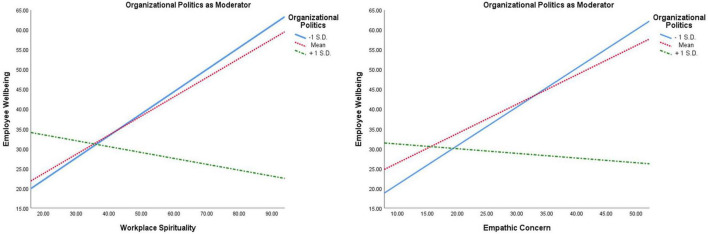
Graphical illustration of the moderation effect of organizational politics on the relationship between workplace spirituality and employee wellbeing; and empathic concern and employee wellbeing.

Moderated mediation analysis (Table 2 and Figure 2) highlighted that organizational politics moderates the path of spirituality and employee wellbeing (β = 0.24; t = 4.26; LLCI = −0.353; ULCI = −0.120 at 95%); and empathic concern and employee wellbeing (β = 0.14; t = 2.66; LLCI = −0.251; ULCI = −0.037 at 95%). The overall structural (Moderated-Mediation) model was found significant on moderated-mediation Index (Index = −0.079; Boot SE = 0.03; LLCI = −0.145; ULCI = −0.013 at 95%).

Organizational politics emerged as a significant moderator in the data analysis, the trends of its moderating effects being depicted in Figure 2. It clearly shows that the level of perception of organizational politics (1 S.D above mean, mean and 1 SD below mean) had notable impact on the effects of workplace spirituality and empathic concern on employee wellbeing. In both cases (workplace spirituality and empathic concern) low level of organizational politics fosters the strength of the effects, but in occasion of average and high level of organizational politics the strength of effects become weak and weaker simultaneously.

## Discussion

Employee wellbeing is a well-researched topic although major section of the literature has focused on factors (e.g., excessive work demands, work stress, inhumane working conditions) those have detrimental influence on employee well-being (Major et al., 2002). Therefore, the positive aspects of work or work environment and how these factors can help in fostering employee wellbeing need sincere attention from the scholars. Previous studies have suggested that job not only provides financial stability but it also fulfills different psychological and social needs of human life (Paul and Batinic, 2010). Hence, it is imperative to understand that work is not always a source of negative experiences, but there are several positive consequences of work climate. It provides employees a meaning in life, opportunity to develop, enhance life satisfaction and many other belongings which are indispensable for the holistic wellbeing. So, there are numerous benefits of positive experiences at workplace and any organization cannot survive for long if it fails to develop positive facets at workplace.

Present study was designed to explore how workplace spirituality and empathic concern impresses betterment of the employees; and whether empathic concern mediate between workplace spirituality and employee wellbeing. Results have supported the hypotheses that workplace spirituality and empathic concern have positive association with employee wellbeing and both these factors contribute largely in determining the status of employee wellbeing (Tables 1, 2). Lyubomirsky et al. (2005) also noted that positive climate of workplace is positively associated with citizenship behavior, altruism, productivity and better performance.

Excessive technological advancement and over-emphasis on profit-making transforms employees as a mean to make money. This approach might help the organizations to attain immediate financial growth but consequently damages the human spirit and psyche of employees. It is not a matter of argument that employees work not only with their head and hand but they need to put their spirit and emotions into work to achieve the organizational objectives (Petchsawang and Duchon, 2009; Chirico et al., 2020). If the employees associate purpose and meaning to their work then they feel spirited into the work. So, workplace spirituality is instrumental in achieving organizational effectiveness and performance (Krahnke et al., 2003; Chirico et al., 2020). Results of this study also showed that workplace spirituality and empathic concern help in fostering employee wellbeing. Present study also established the mediating role of empathic concern between workplace spirituality and employee wellbeing (Figure 1 and Table 2). Diener (1984) advocated that the state of wellbeing not only means the absence of negative experiences but regular occurrence of positive experiences in life. Accordingly, employee wellbeing could not be achieved only by eliminating the negative experiences from the workplace but a sincere look for positive emotional experiences at workplace is also required. Among various positive experiences in human life, spiritual experiences and empathic concern share a unique and special space (Dietz and Kleinlogel, 2014; Chirico and Magnavita, 2019). Empathic concern fosters bonding among individuals and consequently improves the interpersonal relationships, pro-social behaviors and wellbeing of employees (Mencl and May, 2008; Somogyi et al., 2013; Burch et al., 2016). In the organizational context, empathic concern motivates employees to think and engage in welfare of others and improves wellbeing, life satisfaction and performance of the employees (Diener et al., 1999; Scott et al., 2010).

Results of the present research revealed that organizational politics was negatively associated with workplace spirituality and employee wellbeing (Table 1). Kacmar and Baron (1999) also advocated that organizational politics inherently means using immoral or inappropriate tactics to achieve self-centered objectives while ignoring the problems, considerations, values and wellbeing of other employees. By drawing the meaning and motive of organizational politics, the obtained results hardly need any explanation. Previous researches provide ample evidence of often association of organizational politics with negative experiences (such as stress, anxiety and dissatisfaction), deceleration in performance, low job involvement and reduced organizational citizenship behavior (Fandt and Ferris, 1990; Ferris et al., 1996; Chang et al., 2009; Labrague et al., 2017). Substantial numbers of researches have established the adverse effects of organizational politics. However, a dearth of studies focusing on the moderating effects of organizational politics between facilitators of a healthy workplace and employee wellbeing was observed. Present research work attempted to mend existing gap and established how organizational politics moderates the relationship between workplace spirituality, empathic concern and employee wellbeing (Table 2 and Figures 1, 2).

## Conclusion

Present study examined and revealed that workplace spirituality and empathic concern plays a significant role in fostering wellbeing among police personnel. Further, analysis showed that organizational politics negatively affected workplace spirituality and employee wellbeing. Present study also established the role of empathic concern as mediator between employee wellbeing and workplace spirituality. Further, organizational politics was observed to instigate moderating effect between (1) employee wellbeing and empathic concern; and (2) employee wellbeing and workplace spirituality. Present work emphasized need for fundamental shift in approach where organizations should sincerely engage to enhance positivity at workplace rather than merely subsiding negativity. In addition, some studies have indicated that workplace spirituality is also dependent on the nature of work (Crescenzo et al., 2021). Therefore, further studies need to be conducted for examining role of workplace spirituality as a central factor in establishing employee wellbeing.

### Limitations and Future Recommendations

The sample studied was limited to a single organization (Police department) only. Therefore, data from other service organizations (hospital, bank, corporate, etc.) and manufacturing organizations would have encompassed various aspects of work environment and may have provided comprehensive understanding of interactions among research variables. Other compelling variables such as age, work experience and gender were not included in the data analysis. Future studies may include demographic details to identify impact of these variables. A comparative research plan may be executed in the future to identify salient characteristics of diverse work settings and their interactive effect on concerned variables.

## Data Availability Statement

The raw data supporting the conclusions of this article will be made available by the authors, without undue reservation.

## Ethics Statement

Ethical review and approval was not required for the study on human participants in accordance with the local legislation and institutional requirements. The patients/participants provided their written informed consent to participate in this study.

## Author Contributions

SY and PK were responsible for designing the study and data collection. AY, SY, TT, and LM reviewed the literature and drafted the manuscript. TT, AS, and SY completed the data analysis. ND, AY, and PK helped in drafting the final manuscript. All authors significantly contributed to the present research and approved the submission.

## Conflict of Interest

The authors declare that the research was conducted in the absence of any commercial or financial relationships that could be construed as a potential conflict of interest.

## Publisher’s Note

All claims expressed in this article are solely those of the authors and do not necessarily represent those of their affiliated organizations, or those of the publisher, the editors and the reviewers. Any product that may be evaluated in this article, or claim that may be made by its manufacturer, is not guaranteed or endorsed by the publisher.
