# Supplementary figures and images for: Role of Workplace Spirituality, Empathic Concern and Organizational Politics in Employee Wellbeing: A Study on Police Personnel
Source: Front Psychol. 2022 Apr 29;13:881675. doi: 10.3389/fpsyg.2022.881675 (PMC9105451; doi:10.3389/fpsyg.2022.881675)

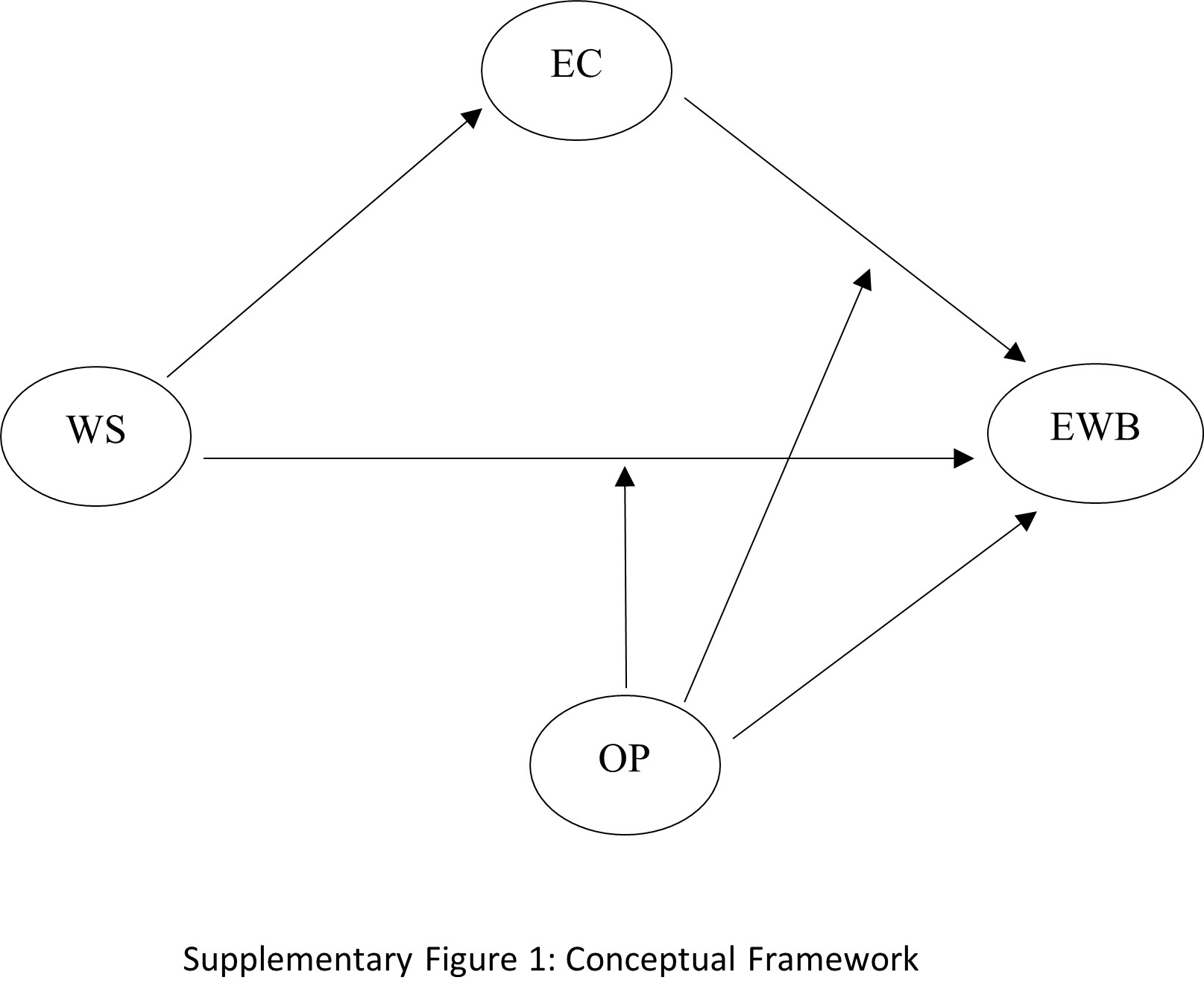

Supplement: Supplementary file 1 [file Image_1.jpeg]
